# A Comparison of Growth Performance, Blood Parameters, Rumen Fermentation, and Bacterial Community of Tibetan Sheep When Fattened by Pasture Grazing versus Stall Feeding

**DOI:** 10.3390/microorganisms12101967

**Published:** 2024-09-28

**Authors:** Huiying Ji, Lili Chen, Yi Ma, Abraham Allan Degen, Zhengrong Yuan, Hualong Chen, Jianwei Zhou

**Affiliations:** 1 Tianjin Key Laboratory of Animal Molecular Breeding and Biotechnology, Tianjin Engineering Research Center of Animal Healthy Farming, Institute of Animal Science and Veterinary, Tianjin Academy of Agricultural Sciences, Tianjin 300381, China; jihy2023@lzu.edu.cn (H.J.); chenlili0609@163.com (L.C.); 2 State Key Laboratory of Herbage Improvement and Grassland Agro-Ecosystems, College of Pastoral Agriculture Science and Technology, Lanzhou University, Lanzhou 730000, China; 220220902161@lzu.edu.cn; 3 Desert Animal Adaptations and Husbandry, Wyler Department of Dryland Agriculture, Blaustein Institutes for Desert Research, Ben-Gurion University of the Negev, Beer Sheva 8410500, Israel; degen@bgu.ac.il; 4 College of Biological Sciences and Technology, Beijing Forestry University, Beijing 100083, China; zryuan@bjfu.edu.cn

**Keywords:** Tibetan sheep, fattening strategies, blood parameters, rumen bacterial community, rumen fermentation

## Abstract

Traditionally, Tibetan sheep only graze pastures without any supplementary feed. However, in recent years, feedlots are being used for fattening Tibetan sheep. The present study compared the growth rates, blood parameters, rumen fermentation, and bacterial communities in Tibetan sheep fattened by pasture grazing (PG) versus those fattened by stall feeding (SF). Twenty 18-month-old Tibetan sheep wethers (42.6 ± 2.11 kg) were divided randomly into PG (n = 10) and SF (n = 10) groups. The PG sheep grazed the grasslands without any supplementary feed, while the SF sheep were offered a commercial total mixed ration (TMR) at a crude protein content of 16.2% DM and an ME of 10.59 MJ/kg. The sheep were on their treatments for 70 days, which included 10 days for adaptation and 60 days for measurements. The average daily gain, white blood cell and lymphocyte counts were greater (*p* < 0.05), while the platelet count was lower (*p* < 0.05) in the SF group than in the PG group. The serum glutathione peroxidase activity, and concentrations of total proteins and albumin were greater (*p* < 0.05), while glucose was lower (*p* < 0.01) in the PG group compared to the SF group. The concentrations of ruminal ammonia–N and total volatile fatty acids (VFAs) were greater (*p* < 0.05), while the pH was lower (*p* < 0.05) in the SF group compared to the PG group. The molar proportion of acetate and the ratio of acetate to propionate were greater (*p* < 0.01) in the PG sheep than in the SF sheep, but the molar proportion of propionate and iso-VFAs did not differ (*p* > 0.05) between the groups. Based on the PCoA, the ruminal bacterial communities were distinct between groups, and the alpha diversity was greater (*p* < 0.001) in the PG sheep than in the SF sheep. The dominant phyla of the rumen bacteria were Firmicutes and Bacteroidetes, while the Firmicutes to Bacteroidetes ratio was greater (*p* < 0.001) in the SF group than in the PG group. At the genus level, the relative abundance of *Ruminococcus* was greater (*p* < 0.05) in the SF group, while the abundances of *Prevotella*, the *Rikenellaceae_RC9_gut_group*, *Butyrivibrio*, and *unclassified_f_Lachnospiraceae* were greater (*p* < 0.05) in the PG group. It was concluded that the Tibetan sheep adopted a short-term intensive fattening strategy when stall fed which altered the rumen bacterial community and blood parameters, enhanced rumen fermentation, and, ultimately, improved their average daily gain.

## 1. Introduction

Tibetan sheep (*Ovis aries*), with a population of 30 million and being one of three original sheep breeds in China [1], inhabit mainly the alpine regions of the Qinghai–Tibetan Plateau (QTP) at an altitude of 3000 to 5000 m [2]. These sheep not only play an important role in maintaining the grassland ecosystem, but also provide Tibetan pastoralists with their main livelihood, meat for food, dung for fuel, and wool for weaving [3]. Traditionally, Tibetan sheep grazed pastures all year without receiving supplementary feed [4], which resulted in a low production efficiency and a slow growth rate of the sheep and impacted the incomes of the herders. In general, Tibetan sheep reach 40 kg at 1.5 years of age. They are fattened on a pasture from June to September and are sold at 50 to 60 kg at 2 to 3 years of age. According to the Feeding Standard of Meat-Producing Sheep and Goats in China [5], the nutrients supplied solely by forages do not meet the requirements for optimal growth for Tibetan sheep, even in the warm season.

With the rapid development of the economy in recent years, Tibetan herders are experimenting with different feeding strategies for Tibetan sheep to improve production and increase profits [6]. For example, modern stall-feeding, named “short-term intensive fattening”, is being used in finishing Tibetan sheep. Because of the difference in nutrition between a natural forage and a concentrate, studies in cattle [7,8], yaks [9], and Eora sheep [10] reported that stall feeding improved the slaughter performance, meat quality, feed utilization efficiency, and profits when compared to livestock grazing solely in a pasture. Stall-fed animals receive all the needed nutrients and experience less stress in foraging for food [11]. 

Ruminants depend mainly on rumen microbes for feed digestion, and ruminal microbial communities are determined primarily by dietary intakes and composition [12]. The energy and protein requirements for ruminants are derived largely from dietary carbohydrate hydrolysis and bacterial protein synthesis through rumen fermentation [13]. In addition, blood parameters, metabolites, and immune capacity indices are commonly used for evaluating the energy balance, nutrient status, and health of an animal. Differences in responses between the grazing and stall-fed cattle [7,8], sheep [14], and yaks [15] have been reported. The concentrate offered to stall-fed animals provides them with the crude proteins and essential elements required to fatten the animals. Such studies comparing grazing and stall feeding have not reported on Tibetan sheep, and this study aimed to fill this important gap. Based on the previous studies [14,15,16], it was reasoned that different feeding regimes could lead to differences in blood parameters and rumen microbial communities in Tibetan sheep on different feeding regimes. Consequently, we hypothesized that (1) growth rate; (2) ruminal bacteria and rumen fermentation; and (3) blood parameters and indices would differ between Tibetan sheep fattened by only grazing and those fattened by stall feeding and would favor the stall-fed sheep. To test these hypotheses, we compared the Tibetan sheep fattened only by grazing and those fattened by stall feeding. In this study, attempts were made to treat the sheep as is done in commercial operations. Therefore, the results of this study should benefit Tibetan herders with references for feeding regimes and improvements in production performance in raising Tibetan sheep on the QPT.

## 2. Materials and Methods

### 2.1. Animals, Diets, and Experimental Design

This study was conducted from July to September 2023 at the Naorigaqi Tibetan Sheep Breeding Cooperatives in Kocai Township, Xiahe Country, Gannan Tibetan Autonomous Prefecture, Gansu Province, China (102°13′24.49″ E, 34°38′12.21″ N, altitude 3260 m above sea level). 

Twenty 18-month-old Tibetan sheep wethers (42.6 ± 2.11 kg) were divided randomly into pasture grazing (PG, n = 10) and stall feeding (SF, n = 10) groups. The PG sheep grazed the rangeland without any supplementary feed following traditional management, while the SF sheep were offered a commercial total mixed ration (TMR). The ingredients and chemical composition of the pasture and the TMR are presented in Table 1. The crude protein level of the TMR was 16.2% and the ME was 10.59 MJ/kg. The experiment was conducted for a total of 70 days from July to September, which included 10 days for adaptation and 60 days for measurements. The SF sheep were maintained in individual pens (80 × 160 cm), were fed ad libitum at 06:30 and 18:30, and had free access to water. The feed offered to the SF sheep was calculated according to their crude protein, energy, and nutrient requirements [5]. Feed in excess was offered so that at least 10% of the feed was left to ensure that the sheep had constant and unrestricted access to the feed. The grazing sheep, at a stocking rate of approximately 3 sheep/ha, had access to a pasture from 06:30 to 20:30 and were corralled overnight. They were rotated between pastures so that a biomass of approximately 217 g DM/m^2^ was available. The dominant plant species were *Kobresia humilis* (Asteraceae), *Kobresia pygmaea* (Cyperaceae), Poa pratensis (Poaceae), Pedicularis kansuensis (Scrophulariaceae), *Elymus nutans* (Poaceae), *Koeleria cristata* (Poaceae), and *Scripustriqueter* (Cyperaceae). The average crude protein level of the pasture was 12.3% DM and the ME was 8.71 MJ/kg (Table 1).

### 2.2. Procedures and Sample Collection

The sheep were weighed before the morning feeding on the first and the 61st day of the treatment to calculate their average daily gain (ADG).

On day 20, 40, and 60, approximately 500 g of fresh mixed forage [18] and 200 g of the TMR diet were collected, thoroughly mixed and placed in self-sealing bags, and stored at −20 °C. On day 61, before the morning feeding, jugular vein blood samples were collected in evacuated tubes: 2 mL in heparinized tubes for the determination of the blood physiological indices and 8 mL in non-heparinized tubes, which were centrifuged at 3000× *g* (4 °C) for 15 min, and the serum was stored at −20 °C. In addition, on day 61 before the morning feeding, 150 mL of rumen fluid was collected from each sheep via an oral stomach tube (Anscitech Co., Ltd., Wuhan, China). The tube was cleaned thoroughly between sample collections, and the first 50 mL was discarded to minimize saliva contamination. The pH of the rumen fluid was measured immediately using a pH meter (PB-10, Sartorius Co., Göttingen, Germany), and then the fluid was filtered through 4 layers of cheesecloth. A total of 5 mL of filtrate was mixed with 5 mL of deproteinizing solution (100 g metaphosphoric acid and 0.6 g crotonic acid per liter) for an analysis of the volatile fatty acids (VFAs); 5 mL of filtrate was mixed with 5 mL of hydrochloric acid solution (0.5 mmol/L) for the ammonia–N measurement; and the remainder was stored at −80 °C for a bacterial analysis.

### 2.3. Chemical Analysis of the Grazed Pasture and Total Mixed Ration

The pasture and TMR samples were dried in a forced-air oven at 65 °C for 72 h, and ground through a 1 mm sieve (JFSO-100, Topu Yunnong Instrument, Hangzhou, China). The DM (method 925.45), organic matter (method 942.05), and ether extract (method 920.29) were determined according to the Association of Official Analytical Chemists [18]. The ether extract (EE; method 920.29) measurement used a reflux system (Ankom XT 15, Fairport, NY, USA), with the petroleum ether at 90°C for 1 h [19]. The total nitrogen (N) content was determined using a nitrogen analyzer (K1100, Hannon Instruments, Jinan, China), and crude protein (CP) content was calculated as the total N content × 6.25. The neutral detergent fiber (aNDF) and acid detergent fiber (ADF) contents were determined by an automatic fiber analyzer (Ankom Technology, Fairport, NY, USA) according to Robertson and Van Soest [20] and Van Soest et al. [21], respectively. 

### 2.4. Analysis of Blood Parameters

Blood measurements were determined using a fully automated blood cell analyzer (BC-5000, Shenzhen), which provided the following: white blood cells (WBCs), neutrophils (NEUTs), lymphocytes (LYMs), red blood cells (RBCs), hemoglobin (HGB), hematocrit (HCT), mean corpuscular volume (MCV), mean hemoglobin (MCH), mean hemoglobin concentration (MCHC), red blood cell distribution width (R-SD), platelets (PLTs), mean platelet volume (MPV), platelet distribution width (PDW), and platelet thrombocythemia (PCT).

Concentrations of the serum albumin (ALB), globulin (GLB), total protein (TP), blood urea nitrogen (BUN), triglycerides (TGs), β-hydroxybutyric acid (BHBA), and glucose (GLU) were determined using an automatic biochemistry analyzer (Hitachi 7160, Hitachi High-Technologies Corporation, Tokyo, Japan), following the protocols of commercial kits (Hunan Fengrui Biotechnology Co., Ltd., Changsha China). The serum growth hormone (GH) concentration was determined using a commercial enzyme-linked immune sorbent assay (ELISA) kit (BYE98273, Shanghai Bangyi Biotechnology Co., Ltd., Shanghai, China). The lowest detectable concentration of the kit was 0.1 ng/mL, and it did not cross-react with other soluble structural analogues. Superoxide dismutase (SOD) activity, malondialdehyde (MDA) content, total antioxidant capacity (T-AOC) and glutathione peroxidase (GSH-Px) activity were determined by kits (Hunan Fengrui Biotechnology Co., Ltd., Changsha, China), following the manufacturer’s instructions.

### 2.5. Determination of Rumen Fermentation Parameters

Concentrations of ruminal VFAs were measured by gas chromatography (GC) using a capillary column (AT-FFAP: 30 m × 0.32 mm × 0.5 mm) in a Shimadzu 2010 plus system (Shimadzu Corporation, Kyoto, Japan), following Liu et al. [22]. The temperature of the injector was 200 °C and of the detector was 250 °C. The program started at an initial temperature of 90 °C, increased to 120 °C at a rate of 10 °C/min, held at 120 °C for 3 min, increased from 120 to 180 °C at a rate of 10 °C/min, and held for 5 min. Ruminal ammonia–N was analyzed following Hristov et al.’s [23] method, using a spectrometer (SpectraMax M5, Molecular Devices, San Jose, CA, USA) at an absorbance of 630 nm.

### 2.6. DNA Extraction, 16S rRNA Gene Amplification, and Sequencing

After the rumen fluid was thawed on ice, the total genomic DNA of the rumen bacteria was extracted using the E.Z.N.A^®^ kit (Omega Bio-tek, Norcross, GA, USA), based on the manufacturer’s instructions. The concentration and purity of the extracted DNA were verified by the 260/280 nm ratio (1.8 to 2.2) using a NanoDrop 2000 UV–vis Spectrophotometer (Thermo Scientific, Wilmington, DE, USA). The quality of the DNA was tested using 1% agarose gel electrophoresis. 

The conventional polymerase chain reaction (PCR) amplification and bioinformatic analysis of the extracted DNA samples were performed by Shanghai Majorbio Bio-Pharm Technology Co., Ltd. (Shanghai, China). The V3-V4 variable regions of the 16S rDNA gene were amplified using primers pair 338F (5′-ACTCCTACGGGAGGCAGCAG-3′) and 806R (5′-GGACTAC HVGGGTWTCTAAT-3′). The bacterial 16S amplification and the quality-filter, cluster, and analysis of the 16S rRNA sequencing data followed the Liu et al. (2019) method. The PCR product was extracted from a 2% agarose gel and purified using the AxyPrep DNA Gel Extraction Kit (Axygen Biosciences, Union City, CA, USA), according to the manufacturer’s instructions, and quantified using a QuantusTM Fluorometer (Promega, Madison, WI, USA).

After amplification, purified amplicons were pooled equimolarly and paired-end sequenced (2 × 300 bp) on an Illumina MiSeq PE300 platform (Illumina, San Diego, CA, USA) by Majorbio Bio-Pharm Technology Co., Ltd. (Shanghai, China). The raw 16S rDNA gene sequencing reads were demultiplexed and quality-filtered by Trimmomatic and merged by FLASH (version 1.2.7) according to the following criteria: (1) the 300 bp reads were truncated at any site receiving an average quality score of <20 over a 50 bp sliding window, and reads shorter than 50 bp or containing ambiguous characters were discarded; (2) only overlapping sequences longer than 10 bp were assembled according to their overlapped sequence. The maximum mismatch ratio of the overlap region was 0.2. Reads that could not be assembled were discarded; (3) individual samples were distinguished according to the barcode and primers in primer matching.

### 2.7. Statistical Analyses

All data were processed using Excel version 2021 and analyzed using SPSS version 26.0 (SPSS Inc., Chicago, IL, USA). Body weight changes, blood parameters, and rumen fermentation variables were compared using a t-test. Statistical significance was accepted at *p* < 0.05, and the results are presented as means and standard error of the mean (SEM). 

The alpha diversity (observed operational taxonomic units—OTUs) for the rumen bacterial community between treatments was calculated with the QIIME (Version 1.9.1) and analyzed by the non-parametric Kruskal–Wallis test and Wilcoxon rank test using the R package. A constrained principal coordinate analysis (PCoA) was used to visualize the classical multi-dimensional scaling of the Bray–Curtis distance matrices, the capscale and anova.cca functions of the vegan package in R (version 3.4.1, USA), and the *p* value were calculated by permutation tests. Spearman’s rank correlation tested the relationships between the relative abundances of the top 20 abundant ruminal bacteria (at genus level) and the fermentation parameters (VFA concentration) using the “corrplot” package in R (version 3.4.1, USA). A linear discriminant analysis effect size (LEfSe) analysis determined the difference in ruminal bacteria between the different fattening strategies by coupling the Kruskal–Wallis test for statistical significance with additional tests assessing biological consistency and effect relevance. Taxa with an LDA score > 4 were considered to have a significant effect size.

## 3. Results

### 3.1. Body Weight Changes

The initial bodyweight (BW) did not differ between sheep groups, while the final BW and average daily gain (ADG) were greater (*p* < 0.05) in the SF group than in the PG group (Table 2). The ADG of the SF sheep was 58.5% greater than that of the PG sheep.

### 3.2. Blood Physiological Measurements

The blood physiological measurements of Tibetan sheep are presented in Table 3. The WBCs and LYMs were greater (*p* < 0.05) in the SF group than in the PG group, while the NEUTs did not differ (*p* > 0.05) between groups. The HCT, MCV, and RS-D were greater (*p* < 0.05) in the PG group than in the SF group, while there was no difference (*p* > 0.05) in the RBC count, HGB, the MCH, and the MCHC between groups. The PLT count was lower (*p* < 0.05) in the SF group than in the PG group; however, the other blood platelet measurements, including the MPV, PCT, and PDW did not differ (*p* > 0.05) between groups. 

### 3.3. Serum Biochemical Metabolites and Antioxidant Capacity

The serum concentrations of TP and ALB were greater, while the GLU was lower (*p* < 0.05; Table 4) in the PG group than in the SF group. The concentrations of GH, GLB, BUN, TG, and BHBA did not differ between groups (*p* > 0.05). The serum concentration of GSH-PX was greater (*p* < 0.05) in the PG sheep than in the SF sheep, but MDA, SOD, and T-AOC did not differ (*p* > 0.05) between groups.

### 3.4. Rumen Fermentation Parameters

The ruminal ammonia–N and total VFAs concentrations were greater (*p* < 0.05), while the pH was lower (*p* < 0.05) in the SF group than in the PG group (Table 5). The molar proportion of acetate was greater, while the proportion of butyrate was lower (*p* < 0.01) in the PG group than in the SF group, but the molar proportions of propionate and iso-VFAs did not differ (*p* > 0.05) between groups. The ratio of acetate to propionate was greater (*p* < 0.01) in the PG group than in the SF group.

### 3.5. Collective Sequencing Data

**Figure 1:**
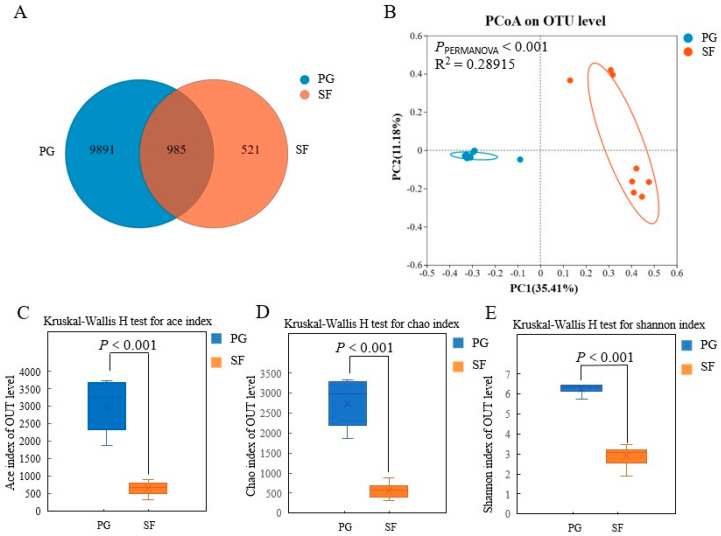
Effects of different fattening strategies on α and β diversity of the ruminal bacterial community in Tibetan sheep. (**A**) Venn graph of OUTs; (**B**) PCoA of the rumen bacteria; (**C**) Ace index; (**D**) Chao index; (**E**) Shannon index.

A total of 11,397 OTUs were obtained based on the 97% nucleotide sequence identity analysis on the processed reads from the rumen fluid samples of the Tibetan sheep (Figure 1). There were 985 OTUs shared between the two groups, and the specific OTUs in the PG and SF groups were 9898 and 521, respectively. The PCoA revealed the differences in the rumen bacterial communities between the PG and SF groups (*p* < 0.01), and a 35.4% and 11.2% distance in PC1 and PC2, respectively. The alpha diversities of the rumen bacteria, including Ace, Chao 1, and Shannon indices, were greater (*p* < 0.001) in the PG group than in the SF group.

### 3.6. Bacterial Community Composition in the Rumen

**Figure 2:**
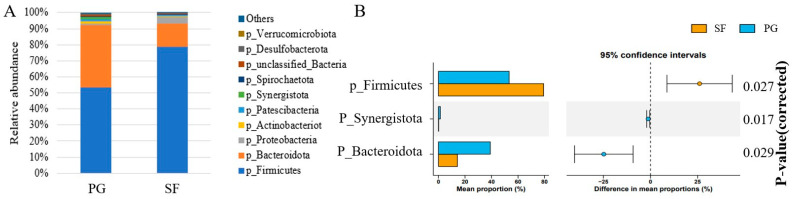
Rumen bacteria classification at phylum level in Tibetan sheep under different fattening strategies. (**A**) Relative abundance; (**B**) Differential rumen bacterial species.

A total of 27 ruminal bacterial phyla were identified in the Tibetan sheep, of which 10 phyla had a relative abundance above 0.5% (Figure 2). The dominant phylum was Firmicutes, with a relative abundance of 53.2% in the PG sheep and 79.3% in the SF sheep; whereas, Bacteroidetes was second, with a relative abundance of 39.1% in the PG sheep and 14.3% in the SF sheep (*p* < 0.01). The ratio of Firmicutes to Bacteroidetes was greater (*p* < 0.01) in SF than PG group, and the relative abundance of Synergistota was greater (*p* < 0.05) in the PG group than in the SF group [Table S1].

**Figure 3:**
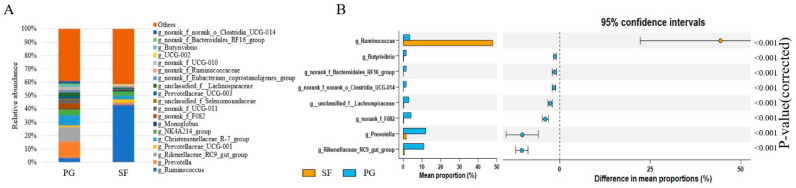
Rumen bacteria classification at genus level in Tibetan sheep under different fattening strategies. (**A**) Relative abundance; (**B**) Differential rumen bacterial species.

In total, 529 bacterial genera were identified, and the top twenty are presented in Figure 3. The most abundant genera were *Ruminococcus*, *Prevotellaceae_UCG-001*, *Prevotella*, the *Christensenellaceae_R-7_group,* and the *Rikenellaceae_RC9_gut_group*. The relative abundance of *Ruminococcus* was greater (*p* < 0.05) in the SF group than in the PG group, but the abundance of *Prevotella,* the *Rikenellaceae_RC9_gut_group*, *norank_f_F082*, *unclassified_f_Lachnospiraceae*, the *Butyrivibrio*, *norank_f_norank_o_Bacteroidales_RF16_group,* and *norank_f_norank_o_Clostridia_UCG-014* were greater (*p* < 0.05) in the PG group than in the SF group [Table S2].

**Figure 4:**
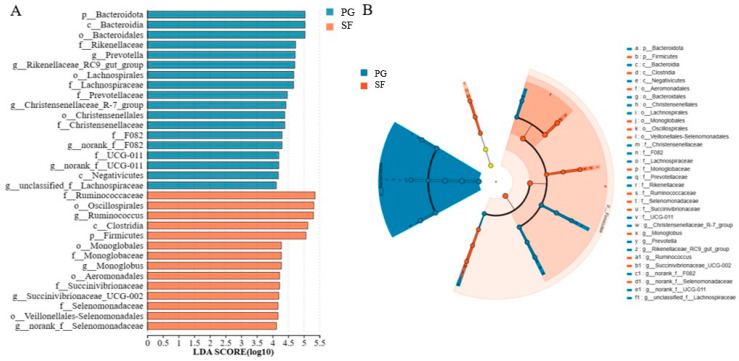
Linear discriminant analysis effect size (LEfSe) for rumen microbiota in Tibetan sheep with different fattening strategies. (**A**) Linear discriminant analysis. (**B**) Cladogram. Prefixes represent abbreviations for the taxonomic rank of each taxon, phylum (p_), class (c_), order (o_), family (f_), and genus (g_).

Differential rumen bacteria that varied between treatments were further identified using linear discriminant analysis effect size (LEfSe; Figure 4). With a default LDA cutoff of ±4.0, differential taxa totaled at 18 genera in the PG sheep and 14 genera in the SF sheep. *Bacteroidaceae*, *Rikenellaceae*, and *Prevotellaceae* families in the SF group, and *Ruminococcaceae*, *Oscillospiraceae*, *Monoglobaceae*, *Succinivibrionaceae*, and *Selenomonadaceae* families in the PG group had high impacts on the differences between these two groups. Cladograms were generated from the LEfSe analysis to illustrate the phylogenetic distribution from class to genus level. The size of each circle in the cladogram represents the abundance of the taxa.

### 3.7. Correlations between Ruminal Bacteria and Fermentation Parameters

**Figure 5:**
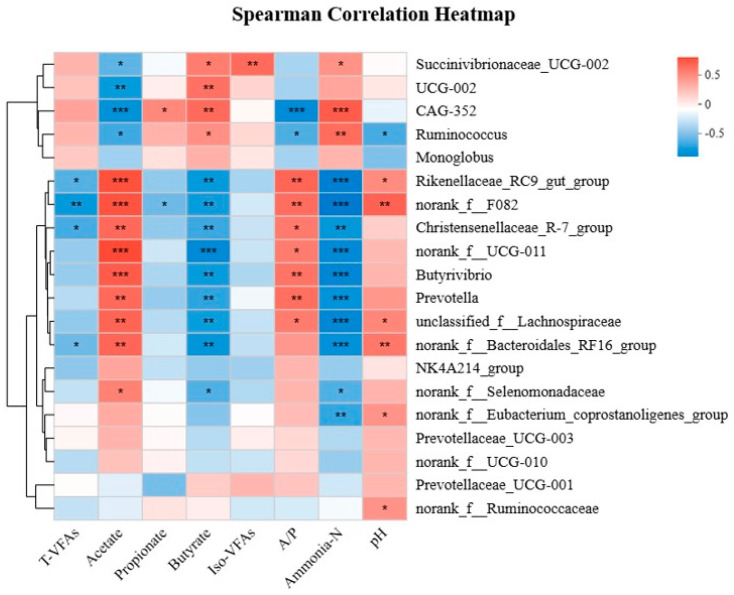
Spearman’s rank correlation analysis between the bacteria at the genus level and rumen fermentation parameters. * *p* < 0.05, and ** *p* < 0.01 *** *p* < 0.001 according to the Spearman’s rank correlation coefficient.

Based on the Spearman rank correlation analysis, 26 positive (*p* < 0.05) and 35 negative (*p* < 0.05) correlations emerged between the relative abundances of the bacterial genera and rumen fermentation parameters (Figure 5). *Ruminococcus* was correlated positively with the concentration of total VFAs (r = 0.592; *p* = 0.006), the concentration of ammonia–N (r = 0.552; *p* = 0.012), and the molar proportion of propionate (r = 0.448; *p* = 0.048). *Prevotella* was correlated positively with the molar proportion of acetate (r = 0.635; *p* = 0.003) and the A:P ratio (r = 0.522; *p* = 0.018), and negatively with the concentration of total VFAs (r = −0.502; *p* = 0.024) and ammonia–N (r = −0.626; *p* = 0.003) and the molar proportion of butyrate (r = −0.544; *p* = 0.013). *Rinenellaceae_RC9_gut group* was correlated positively with the molar proportion of acetate (r = 0.678; *p* = 0.001) and the A:P ratio (r = 0.605; *p* = 0.005), and negatively with the concentrations of total VFAs (r = −0.583; *p* = 0.007) and ammonia–N (r = −0.737; *p* < 0.001) and the molar proportion of butyrate (r = −0.5736; *p* = 0.008). *Butyrivibrio*, *Unclassified_f_Lachnospiraceae*, *Christensenellaceae_R-7_group*, *norank_f_F082*, and *norank_f_norank_o_Clostridia_UCG-014* were correlated positively with the molar proportions of acetate and the A:P ratio, and negatively with the molar proportion of butyrate and the concentration of ammonia–N.

## 4. Discussion

### 4.1. Effect of Different Fattening Strategies on Blood Parameters Measurements

Blood parameters reflect the nutrient metabolism, physiological activities, and health status of an animal. WBCs, consisting of monocytes, neutrophils, eosinophils, and lymphocytes, play an important role in healing bodily injuries, resisting pathogen invasion, and integrating immune responses [24]. Previous studies reported a greater WBC count in stall-fed livestock than in grazing livestock [14,25], which was also observed in the present study. In addition, Liu et al. [26] reported that the number of WBCs increased with stress in weanling yak calves. These results indicate that the stall-fed sheep were more stressed than the grazing sheep, perhaps due to the change in management and the restricted movement in the pens. 

The RBCs, HGB, and the HCT in livestock are associated with the capacity of oxygen and carbon dioxide transportation [27]. The RBC is the main medium for blood oxygen transport, while HGB is the oxygen carrier. Previous studies reported that the RBC count, HGB concentration and HCT in the blood of Tibetan sheep and yaks were greater than that of lowland ruminants, and these blood variables increased with altitude [28]. An increase in these parameters enables more oxygen to be transported to tissue, which is important at high altitudes because of the decrease in air oxygen content. In the present study, the RBC and HGB in both groups were approximately 11.4 × 1012/L and 150 g/L, respectively, which were greater than the values reported in the lowland sheep [29]. The MCV and R-SD reflect the size of the RBCs, and the high MCV and R-SD indicate large RBCs and an increased RBC surface, thus facilitating oxygen transportation. The slightly greater HCT, MCV, and R-SD in the PG group compared to the SF group suggests that grazing Tibetan sheep require more oxygen for their foraging. 

Blood PLTs play an important role in coagulation and hemostasis [30], and are involved in the regulation of physiological responses, such as inflammatory reactions, immune responses, and cold resistance [31]. The blood PLT count increased in sheep [32] and cows [33] after cold exposure; whereas, Liu et al. [25] observed that the count was greater in grazing sheep than in stall-fed sheep, as was observed in the present study. More research is warranted to explain these differences.

### 4.2. Effect of Different Fattening Strategies on Serum Metabolites and Antioxidant Capacity

Serum metabolites and hormones are considered to be key indicators of physiological functions, nutritional utilization, and health status of livestock [34]. The serum TP is composed mainly of ALB and GLB, with approximately 35% to 50% consisting of ALB. ALB plays an important role in homeostasis and osmolality maintenance, nutrient transport, and free radical clearance, while GLB is involved in the regulation of inflammatory reactions and pathological injury resistance [34]. Protein is the largest component in the solid part of the serum, and is generally used to assess whole-body protein metabolism, the functions of liver protein synthesis, and kidney protein filtration [35]. It was reported that animals with a faster growth rate had a greater concentration of serum TP [36], and that the concentration of TP was greater in the stall-fed yaks than in the grazing yaks [37]. The TP and ALB concentrations were greater in the PG sheep than in the SF sheep in the present study. A greater serum ALB was associated with a better capacity for scavenging free radicals [38]. Therefore, we reasoned that the greater serum ALB in the PG sheep than in the SF sheep was probably caused by a greater need to eliminate free radicals. The serum GSH-PX activity data supported this premise. 

BUN is the end metabolite of protein, peptides, and amino acid metabolism, and was strongly correlated with the dietary protein concentration [39]. The serum BUN concentration reflects the balance of dietary amino acids and utilization efficiency of the body protein [40]. In the present study, the dietary protein concentration was greater in the SF sheep than in the PG sheep, while the serum BUN concentration was similar between these two treatments, which suggested the SF sheep utilized the dietary proteins more efficiently. In the ruminants, the serum GLU concentration is a key indicator of energy balance, and propionate is the precursor of GLU. It was reported that livestock with a higher serum GLU concentration would have a greater growth performance [4], which was in line with the results of the present study. The greater serum GLU concentration in the SF group compared to the PG group was mainly attributed to the greater ruminal absolute propionate production in the SF sheep. BHBA is a main ketone substrate in blood, and is generally used to assess the energy balance in livestock [41]. The serum BHBA concentration increases with a negative energy balance, and a threshold value above 1.0 mmol/L is considered an insufficient energy supply for the ruminants [42]. In the present study, the serum BHBA concentration in the PG sheep was close to the threshold, and was numerically greater than that in the SF sheep, which indicated that the dietary energy balance of the stall-fed sheep was better than the grazing sheep. 

The GSH-Px is an antioxidant enzyme that scavenges peroxides and free radicals generated in the body, and not only protects cells from injury by the free radicals, but also repairs cell membranes that have undergone oxidation [43]. In the present study, the activity of the serum GSH-Px was greater in the grazing sheep than in the stall-fed sheep, which was consistent with the findings in yaks [25]. Free radical production increases with exercise, and as the PG sheep moved more than the SF sheep, they should produce more free radicals. This could explain the greater serum activity of GSH-Px in the PG sheep compared to the PF sheep [44]. Furthermore, alpine plants are rich in antioxidant components, such as phenols, polyunsaturated fatty acids, and vitamins [45], which could also contribute to the greater serum GSH-Px activity in the grazing Tibetan sheep.

### 4.3. Effect of Different Fattening Strategies on Rumen Fermentation Parameters

The optimal pH for rumen fermentation ranges between 6.2 and 7.2, while fibrolytic bacteria are inhibited at a pH below 6.2 [46]. In the present study, the ruminal pH was 7.22 for the PG sheep and 6.96 for the SF sheep, and both were within the optimal ranges. However, the ruminal pH was lower in the SF sheep than in the PG sheep, which was mainly due to the greater amount of dietary non-fibrous carbohydrates (NFCs) consumed by the SF sheep. The NFCs are degraded rapidly and converted to VFAs by the rumen microbes, which decreases the ruminal pH. Ammonia–N is the major nitrogen source for ruminal microbial protein synthesis, and accounts for 40% to 68% of the total crude requirement [47]. It was reported that the optimal ruminal ammonia concentration for microbial growth ranges between 5 and 25 mg/dL [48]. In the present study, the ruminal ammonia–N concentrations of the PG and SF sheep were 6.09 and 16.9 mg/dL, respectively, which fell within the optimal ranges. However, the ruminal ammonia–N concentration was greater in the SF sheep than in the PG sheep, which was likely a result of the higher protein content in the total mixed ration than in the grazed forage.

The VFAs, consisting mainly of acetate, propionate, and butyrate, are the end metabolites of carbohydrate degradation by the rumen microbes, and can supply the host ruminant with 70% to 80% of its energy needs [49]. Dietary composition and nutrient content affect rumen fermentation [50]. For example, a high fiber forage diet promotes acetate production [51]; whereas a high concentrate diet that is high in NSCs enhances propionate and butyrate production [52,53]. This could explain the greater molar proportion of acetate in the PG sheep and the greater molar proportion of butyrate in the SF sheep. In the reactions of acetate and propionate generated from the pyruvate, hydrogen, a key precursor in methane synthesis, is released in the acetate pathway and is incorporated into the propionate pathway. The ratio of acetate to propionate was greater in the PG sheep than in the SF sheep, which suggests that the efficiency utilization of dietary energy was better in the stall-fed sheep than in the grazing sheep. 

### 4.4. Effect of Fattening Strategy on Rumen Bacterial Diversity and Bacterial Community Composition

Rumen bacterial communities play a key role in the maintenance of productivity, immunity, health, and survival of the host. Generally, the diversity and structure of the rumen bacterial community are strongly associated with dietary composition, feeding regimes, environmental factors, animal species, and host genetics [12,54,55]. An increase in diversity enhances the stability of the rumen bacterial community, and improves the feed utilization efficiency [56]. In the present study, alpha diversity indices, including ACE, Chao, and Shannon, were all greater in the PG group than in the SF group, most likely because the grazing sheep consumed a wide variety of forages while the stall-fed sheep consumed a stable diet [57]. Guo [58] recorded hundreds of forage species on the QTP grassland. In addition, the harsh alpine environment induces protective secondary compounds in the forages, such as tannins, waxes, and flavonoids [59], which increase the bacterial community diversity in the grazing ruminants [60]. The PCoA revealed that the beta diversity of the bacterial composition and structure were distinct between the PG and SF groups, which was in agreement with a previous study [61], indicating that feed is an important factor affecting the rumen bacterial community.

Consistent with previous studies on ruminants [62,63,64], the most dominant bacteria phyla in the rumen of the Tibetan sheep in the present study were Firmicutes and Bacteroidetes. Firmicutes plays an important role in the degradation of fiber, cellulose, starch, and oligosaccharides [65], and, consequently, is closely related to energy metabolism. Bacteroidetes are important in the degradation of carbohydrates and proteins, and in the development of the immune system in the gut. It was reported that the Firmicutes–Bacteroidetes ratio reflects the feed conversion efficiency, energy utilization and growth performance [66]. Zou et al. [67] reported that grazing yaks with a low Firmicutes–Bacteroidetes ratio displayed an inefficient feed efficiency; whereas Liu et al. [68] observed that the Firmicutes–Bacteroidetes ratio increased linearly with an increase in the dietary energy level, and correlated positively with body weight gain. The Firmicutes–Bacteroidetes ratio in the present study was greater in the SF sheep than in the PG sheep, which suggested that the stall-fed sheep had a greater feed conversion efficiency than the grazing sheep, which supports, at least in part, the greater ADG in these sheep. The phylum Synergistota has a mutual symbiotic relationship with methanogens [69]. A higher relative abundance of Synergistota was observed in the PG sheep compared to the SF sheep, indicating that the grazing Tibetan sheep emit more enteric methane and have a lower efficiency of dietary energy utilization. This was in accordance with the earlier discussion on the acetate to propionate ratio.

The genus *Prevotella* plays an important role in the degradation of dietary proteins, starch, and hemicellulose [70] and is involved in fiber decomposition [71]. Consequently, *Prevotella* is considered a bacteria possessing a wide functional versatility [72]. In general, *Prevotella* was the most dominant genus in steers [60], dairy cows [73], and goats [74] when consuming high quality diets; however, *Prevotella* was more abundant in the PG sheep than in the SF sheep in the present study. The reason was probably due to the high concentration of polyunsaturated fatty acids in the forages (approximately 40%) consumed by the PG sheep. *Prevotella* increased with the supplementation of plant and marine oil in an in vitro study [75]. Furthermore, it was reported that the relative abundance of *Prevotella* decreased linearly with an increasing energy level [68,76], which could explain the lower abundance of *Prevotella* in the SF group than in the PG group. In the present study, *Prevotella* was correlated positively with acetate and negatively with the total VFA, butyrate, and ammonia–N concentrations.

*Ruminococcus* is commonly regarded as a fibrolytic bacteria, and is predominantly present in the gastrointestinal tract of ruminants consuming high-fiber diets [73]. This genus is involved in the production of acetate [77]. However, in the present study, the relative abundance of *Ruminococcus* was greater in the SF sheep than in the PG sheep, and was correlated positively with the concentrations of propionate and total VFAs. It was reported that *Ruminococcus* displayed high amylolytic and lipolytic activities [78], and the SF diet, rich in starch and fat, most likely stimulated the growth and proliferation of this bacteria [79]. In addition, *Ruminococcus* was associated with the absorption of nutrients [80], immune regulation, and health maintenance [81] of the intestines of the host animals. The relative abundance of *Ruminococcus* was greater in the SF group than in the PG group, which indicated that the stall- fed sheep received adequate nutrients, and maintained the stability and health of the rumen internal environment.

The *Rikenellaceae_RC9_gut_group* was a dominant genus in the rumen in the present study, which was consistent with previous studies [62,82]. It was reported that the *Rikenellaceae_RC9_gut_group* was involved in the degradation of plant-derived polysaccharides, such as cellulose and hemicellulose, and acetate was the predominant end product [83]. This could explain the positive correlation between the relative abundance of the *Rikenellaceae_RC9_gut_group* and the ruminal acetate concentration in the present study. The relative abundance of the *Rikenellaceae_RC9_gut_group* was greater in the PG sheep than in the SF sheep, which was attributed to the greater NDF and ADF contents in the forages, and resulted in a greater molar proportion of acetate in the PG group. Similarly, the genera *Butyrivibrio* and *Unclassified_f_Lachnospiraceae*, both fibrolytic bacteria [84,85], were greater in the PG sheep than in the SF sheep and were correlated positively with the ruminal acetate concentration. *Clostridia_UCG-014*, commonly regarded as a pro-inflammatory bacterium [86], was greater in the PG sheep than in the SF sheep, indicating possible greater inflammation in the grazing sheep. The negative correlation between *Clostridia_UCG-014* and the butyrate concentration supported our reasoning, as butyrate has a key role in maintaining the health of the gut [87].

The relative abundance of the genus *RF_16_group*, belonging to the phylum Bacteroidetes, was greater in the grazing sheep than in the stall-fed sheep, which was in accordance with the previous studies on yaks [88]. Moreover, this genus was correlated positively with the ruminal acetate concentration and negatively with the total VFAs, propionate, and butyrate concentrations, indicating that the relative abundance of the *RF_16_group* might be associated with fiber degradation. It was reported that the genus *norank_f_F082* played an important role in dietary NFC degradation, which was increased with the increasing levels of NFC in the dietary concentrates [89]. Interestingly, the relative abundance of *norank_f_F082* was lower in the SF group than in the PG group, even though the SF diet was rich in NFC content, which implied that the *norank_f_F082* bacteria possessed certain unrecognized functions.

The LEfSe analysis revealed that the main bacteria biomarkers in the PG group were the families *Bacteroidaceae, Rikenellaceae*, and *Prevotellaceae*, all belonging to the Bacteroidetes phylum. The relative abundance of ruminal Bacteroidetes increased with the dietary fiber content and the ruminal Bacteroidetes were involved in fiber degradation [90]. The main bacteria biomarkers in the SF group were the families *Ruminococcaceae*, *Oscillospiraceae*, *Monoglobaceae*, *Succinivibrionaceae*, and *Selenomonadaceae*, and they all belonged to the Firmicutes phylum. Firmicutes was reported to be associated with intensive energy metabolism and high feed utilization [79,91,92]. In the present study, the relative abundance of Firmicutes was greater in the SF group than in the PG group, suggesting that the stall-fed Tibetan sheep were supplied with energy-rich diets, and displayed greater efficiency in feed utilization, which increased their ADG.

## 5. Conclusions

The concentrations of serum glucose and ruminal total VFAs were greater in the SF sheep than in the PG sheep, which indicated the stall-fed Tibetan sheep consumed adequate dietary energy, and resulted in a greater ADG. The alpha diversity of the ruminal bacteria was greater in the PG sheep than in the SF sheep, and the beta diversity revealed that the bacterial communities were separated between treatments, which implied different rumen bacterial communities between the sheep groups. The relative abundance of the amylolytic bacteria *Ruminococcus* was greater in the SF group; whereas the fibrolytic bacteria, such as the *Rikenellaceae_RC9_gut_group*, *Unclassified_f_Lachnospiraceae*, and *Butyrivibrio*, were lower in the SF group than in the PG group, which resulted in a greater molar proportion of acetate in the grazing Tibetan sheep. In conclusion, the Tibetan sheep adapted to stall feeding by altering the rumen bacterial community and blood parameters, enhancing rumen fermentation and increasing their average daily gain when compared with traditional grazing management.

## Figures and Tables

**Table 1:** Ingredients and chemical composition of the grazed pasture and total mixed ration.

Items	Pasture	Total Mixed Ration
**Ingredient (air-dried basis), g/kg DM**		
Corn straw	-	200
Sunflower seed hull	-	50.0
Corn grain	-	220
Corn bran	-	135
Corn germ meal	-	170
Soybean meal	-	20.0
Cottonseed meal	-	30.0
Palm meal	-	40.0
Molasses	-	30.0
Corn sugar residue	-	80.0
Limestone	-	14.0
NaCl	-	5.70
Premix ^1^	-	5.30
**Chemical compositions, g/kg DM basis**		
DM	310	911
OM	935	920
CP	123	162
Ether extract	39	42
aNDF	477	348
ADF	305	123
ME ^2^, MJ/kg	8.71	10.59

DM, dry matter; OM, organic matter; CP, crude protein; aNDF, neutral detergent fiber; ADF, acid detergent fiber; ME, metabolizable energy. ^1^ The premix provided the following per kg for diets: Fe 25 mg, Mn 40 mg, Zn 40 mg, Cu 8 mg, I 0.3 mg, Se 0.2 mg, Co 0.1 mg, VA 3300 IU, VD 3600 IU, VE 20 IU. ^2^ The ME was calculated according to the Tables of Feed Composition and Nutritive Values in China as follows: ME (MJ/kg) = 0.82 × DE (MJ/kg) = 0.82 × (0.209 × CP% + 0.322 × EE% + 0.084 × CF% + 0.002 × NFE%2 + 0.046 × NFE% − 0.627) [17].

**Table 2:** Effect of fattening strategies on body weight changes in Tibetan sheep.

Items	Fattening Treatment		SEM	*p*-Value
	PG	SF		
Initial BW, kg	41.6	42.9	1.08	0.182
Final BW, kg	49.1	55.3	1.77	<0.01
ADG, g/d	202.7	321.2	25.5	<0.01

BW, body weight; ADG, average daily gain. PG, pasture grazing; SF, stall feeding; SEM, standard error of the means.

**Table 3:** Effect of fattening strategies on blood physiological measurements in Tibetan sheep.

Items	Fattening Treatment		SEM	*p*-Value
	PG	SF		
**Blood immune cells**				
WBC, ×10^9^/L	7.91	9.71	0.749	0.027
NEUT, ×10^9^/L	2.83	3.28	0.749	0.336
LYM, ×10^9^/L	4.05	5.13	0.490	0.041
**Red blood cells hemoglobin**				
RBC, ×10^12^/L	11.4	11.4	0.285	0.503
HGB, g/L	150	149	3.3	0.953
HCT, %	41.9	38.8	0.92	<0.01
MCV, fL	35.8	34.0	0.40	<0.001
MCH, pg	13.3	13.2	0.18	0.621
MCHC, g/L	371	387	8.8	0.099
R-SD, f/L	23.4	19.9	0.82	<0.001
**Blood platelet**				
PLT, ×10^9^/L	418	317	48.8	0.034
MPV, fL	8.21	8.96	0.571	0.210
PCT, %	0.30	0.31	0.572	0.920
PDW, fL	15.3	15.7	0.23	0.084

WBC, white blood cell; NEUT, neutrophil count; LYM, lymphocyte; RBC, red blood cell; HGB, hemoglobin; HCT, hematocrit; MCV, mean corpuscular volume; MCH, mean hemoglobin; MCHC, mean hemoglobin concentration; RS-D, red blood cell distribution width; PLT, platelet; MPV, mean platelet volume; PCT, platelet hematocrit; PDW, platelet distribution width; PG, pasture grazing; SF, stall feeding; SEM, standard error of the means.

**Table 4:** Effect of different fattening strategies on serum biochemical parameters and antioxidant capacity of Tibetan sheep.

Items	Fattening Treatment		SEM	*p*-Value
	PG	SF		
**Biochemical parameters**				
TP, g/L	72.6	66.7	1.86	<0.01
ALB, g/L	44.9	41.7	1.15	0.013
GLB, g/L	27.3	25.9	1.09	0.246
BUN, mmol/L	9.72	8.97	0.866	0.399
GLU, mmol/L	4.39	6.54	0.586	<0.01
TG, mmol/L	0.29	0.31	0.055	0.705
BHBA, mmol/L	0.96	0.79	0.171	0.318
GH, ng/mL	6.00	6.20	0.919	0.828
**Antioxidant capacity**				
SOD, U/mL	142	127	7.6	0.061
T-AOC, U/mL	4.69	3.80	0.789	0.275
GSH-Px, U/mL	141	107	17.5	0.015
MDA, μmol/mL	3.51	3.99	0.885	0.592

TP, total protein; ALB, albumin; GLB, globulin; BUN, blood urea nitrogen; GLU, glucose; TG, triglyceride; BHBA, β-hydroxybutyric acid; GH, growth hormone; SOD, superoxide dismutase; T-AOC, Total antioxidant capacity; GSH-Px, glutathione peroxidase; MDA, malondialdehyde; PG, pasture grazing; SF, stall feeding; SEM, standard error of the means.

**Table 5:** Effect of different fattening strategies on rumen fermentation parameters of Tibetan sheep.

**Items**	**Fattening Treatment**		**SEM**	***p*-Value**
	**PG**	**SF**		
pH	7.22	6.96	0.112	0.032
Ammonia–N, mg/dL	6.09	16.9	1.20	<0.01
Total VFAs, mM	45.8	55.4	4.40	0.043
**VFA, mol/100 mol**				
Acetate (A)	74.5	59.5	2.14	<0.01
Propionate (P)	15.0	17.0	1.48	0.199
Butyrate	7.47	18.5	1.742	<0.01
Iso-VFA	0.03	0.03	0.003	0.495
A:P	4.98	3.95	0.280	<0.01

VFAs, volatile fatty acids; N, nitrogen. PG, pasture grazing; SF, stall feeding; SEM, standard error of the means.

## Data Availability

The data that support the findings of this study are available on request from the corresponding author. The data are not publicly available due to privacy or ethical restrictions.

## Supplementary Materials

The following supporting information can be downloaded at: https://www.mdpi.com/article/10.3390/microorganisms12101967/s1, Tables S1 and S2 show the levels of phylum and genus in terms of the relative abundance of microorganisms.
