# Supplementary material for: A Comparison of Growth Performance, Blood Parameters, Rumen Fermentation, and Bacterial Community of Tibetan Sheep When Fattened by Pasture Grazing versus Stall Feeding
Source: Microorganisms. 2024 Sep 28;12(10):1967. doi: 10.3390/microorganisms12101967 (PMC11509657; doi:10.3390/microorganisms12101967)
Supplement: Supplementary file 1 [file microorganisms-12-01967-s001.zip › microorganisms-3169812-supplementary.pdf]

Table S1. Composition of dominate ruminal bacterial communities at phylum level (relative abundance > 1.00%)

Phylum	Fattening treatments		SEM	P-value
	PG	SF		
Firmicutes	53.24	79.28	7.913	<0.01
Bacteroidota	39.05	14.27	7.165	<0.01
Proteobacteria	0.79	4.19	3.311	0.319
Actinobacteriota	1.40	0.60	0.392	0.056
Patescibacteria	1.30	0.61	0.484	0.166
Synergistota	1.38	0.15	0.372	<0.01
Spirochaetota	0.55	0.49	0.311	0.834
unclassified_Bacteria	0.93	0.63	0.155	0.051
Desulfobacterota	0.65	0.26	0.216	0.075
Verrucomicrobiota	0.36	0.16	0.118	0.059

Table S2. Composition of dominate ruminal bacterial communities at genus level (relative abundance > 1.00%)

Genus	Fattening treatments		SEM	P-value
	PG	SF		
Ruminococcus	1.71	59.33	7.16	<0.01
Prevotella	11.89	1.5	1.98	<0.01
Rikenellaceae_RC9_gut_group	10.96	0.54	0.78	<0.01
Prevotellaceae_UCG-001	1.14	9.54	5.51	0.161
Christensenellaceae_R-7_group	7.92	2.6	1.44	0.052
NK4A214_group	4.11	2.84	0.95	0.211
Monoglobus	0.39	3.92	2.11	0.128
norank_f_F082	4.12	0.13	0.4	<0.01
norank_f_UCG-011	3.61	0.16	0.87	0.053
unclassified_f_Selenomonadaceae	0.9	0.23	0.26	0.062
Prevotellaceae_UCG-003	1.62	1.57	1.01	0.961
unclassified_f__Lachnospiraceae	2.91	0.24	0.26	<0.01
norank_f_Eubacterium_coprostanoligenes_group	2.02	1.12	0.56	0.125
norank_f_Ruminococcaceae	0.63	2.29	1.85	0.384
norank_f_UCG-010	1.45	0.6	0.29	0.051
UCG-002	0.23	1.77	0.76	0.072
Butyrivibrio	1.54	0.22	0.19	<0.01
norank_f_Bacteroidales_RF16_group	1.57	0.09	0.26	<0.01
norank_f_norank_o_Clostridia_UCG-014	1.6	0.04	0.22	<0.01
